# Un cas de cataracte céruléenne congénitale

**DOI:** 10.11604/pamj.2014.19.112.5423

**Published:** 2014-10-01

**Authors:** Fatima Zohra El Meriague, Rajae Daoudi

**Affiliations:** 1 Université Mohammed V Souissi, Service d'Ophtalmologie A de l'hôpital des spécialités, Centre Hospitalier Universitaire, Rabat, Maroc

**Keywords:** Cataracte, céruléenne, congénitale, cataract, cerulean, congenital

## Image en medicine

Nous rapportons le cas d'un patient de 15 ans, qui consulte pour une baisse de l'acuité visuelle au niveau de l'oeil droit depuis 2 ans. A l'examen clinique, l'acuité visuelle est 1 /10. Le tonus oculaire est à 14 mmhg. L'examen du segment antérieur montre l'existence d'une cataracte faite d'opacités blanchâtres correspondant à une cataracte céruléenne congénitale. Le traitement a consisté en une cure de la cataracte par phacoémulsification avec une bonne évolution. La cataracte céruléenne est une forme de cataracte congénitale rare à teinte bleutée, faite d'opacités blanchâtres en couches concentriques avec en leur centre une disposition radiaire. L'acuité visuelle est assez bonne dans l'enfance mais peut se dégrader ultérieurement. Le gène de type 1 (CCA1) est en 17q24, le gène CRYBB2 de type 2 (CCA2) est en 22q. L'affection est autosomique dominante.

**Figure 1:**
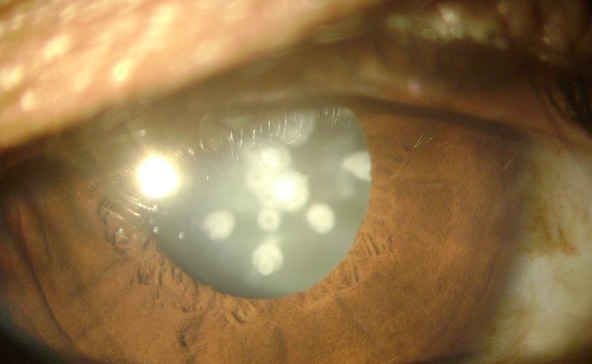
Cataracte céruléenne

